# Disclosure and consent: ensuring the ethical provision of information regarding childbirth

**DOI:** 10.1136/jme-2022-108283

**Published:** 2023-06-12

**Authors:** Kelly Irvine, Rebecca CH Brown, Julian Savulescu

**Affiliations:** 1 University of Melbourne VCCC, Parkville, Victoria, Australia; 2 Oxford Uehiro Centre for Practical Ethics, University of Oxford, Oxford, UK; 3 Faculty of Philosophy, University of Oxford, Oxford, UK; 4 Murdoch Children's Research Institute, Parkville, Victoria, Australia; 5 Centre for Biomedical Ethics, Yong Loo Lin School of Medicine, National University of Singapore, Singapore

**Keywords:** Decision Making, Ethics- Medical, Informed Consent, Women, Child

## Abstract

Ethical medical care of pregnant women in Australia should include the real provision of information regarding the risks and benefits of vaginal birth. Routinely obtaining consent for the different ways in which childbirth is commonly intervened on and the assistance involved (such as midwife-led care or a planned caesarean section) and providing sufficient information for women to evaluate the harms and benefits of the care on offer, would not only enable the empowerment of women but would align with the current standard of care as established by Rogers v Whittaker.

 This paper will consider the respective risks of a trial of labour versus caesarean birth on maternal request. We will argue that women should explicitly give valid consent for their preferred approach to their delivery care—including midwifery assistance—over alternative options (such as elective caesarean section). We outline the current landscape, guidelines and language used regarding caesareans and evaluate whether the current process and focus on providing information and explicitly seeking consent in relation to caesarean section are consistent with ethical medical care. We argue that the current approach to obstetric care is inconsistent with the principles and values demonstrated within the healthcare system. Not informing women of all risks of their birthing options, including a trial of labour, may result from value-based judgements of what is in their interests and limits women’s right to be in control of their treatment and birth.

## Duty to inform

In Australia, the High Court decision of Rogers v Whitaker (1992) 175 CLR 479 established the duty to provide information to patients of risks that are material to the patient,^1^ providing at 489:

Whether the patient has been given all the relevant information to choose between undergoing and not undergoing treatment is a question of a different order. Generally speaking, it is not a question the answer to which depends on medical standards or practices.

Rogers v Whittaker established a new standard of care with respect to the provision of information to patients regarding risks, pertinent and relevant to their circumstances. This precedent deviated from the then existing Bolam test, established by the English decision, which provided that a ‘practitioner is not negligent if he acts in accordance with a practice accepted at the time as proper by a responsible body of medical opinion’.^2 3^

While Rogers v Whittaker changed the practice by which all medical practitioners inform patients of the risks and benefits of treatment and obtain their consent, it is not clear whether this has extended in practice to the provision of information to pregnant women. This is due to the fact that vaginal birth is not, in and of itself, a medical intervention requiring consent.

## Current practice

In Australia, in 2015, 67% of women gave birth vaginally and 33% via caesarean section (including emergency caesarean section).^4^ Guidelines published by The Royal Australian and New Zealand College of Obstetricians and Gynaecologists (RANZOG) make provision for women to request an elective caesarean section (so long as they can demonstrate an understanding of the risks and benefits). However, the language used to discuss maternal request for caesarean sections frequently refers to there being ‘no medical reason’ for caesarean delivery, implicitly indicating the non-necessity of such a procedure.^5^ In a government report, *Australia’s Health*, it is stated: ‘As caesarean section rates increase, it is important that mothers are aware of the risks involved with this procedure, and that caesarean sections should occur only when there is a clinical need’.^4^

Clinical guidelines recognise that caesarean births in the context of ‘significant psychological factors’ such as ‘previous traumatic birth… or significant life trauma… may legitimately be also considered “medically indicated’.^5^

A lack of data means it is unclear how many caesarean sections are performed on maternal request. The Australian Atlas of Health Care Variation for 2015 found that ‘between 42% and 60% of planned caesarean sections performed before 39 weeks’ gestation did not have a medical or obstetric indication’.^6^ Similarly, the risks (and benefits) of elective caesarean sections are poorly understood due to failure to analyse outcomes for those performed on maternal request as opposed to those performed for ‘medically indicated’ reasons (including emergency caesarean sections).

Currently in Australia, it is unclear whether all patients are routinely informed of all risks associated with a trial of vaginal birth. Public health policy in many states does not provide for maternal request caesarean section (MRCS).^7^,^10^ Despite criticism, New South Wales and Queensland still employ ‘normal birth’ policies, which clearly define a birth that does not include a caesarean section.^8^,^11^ In addition to anecdotal suggestions that MRCS is not available consistently in the public health system in Australia, in the absence of an express positive obligation in public health guidelines and policy, it cannot be assumed that all pregnant women in Australia have a choice. As a result, it is not too long a bow to infer that public health policies designed to promote increasing vaginal birth rates are unlikely to foster the provision of fulsome and unbiased information regarding the risks of vaginal birth or their associated interventions.^12^

The absence of express policy in Australia regarding caesarean section on maternal request is in contrast with the express policy contained in the United Kingdom’s NICE (National Institute for Health and Care Excellence) Clinical Guidelines on Caesarean birth.^13^ The NICE Guidelines provide:

If a vaginal birth is still not an acceptable option after discussion of the benefits and risks and offer of support (including perinatal mental health support if appropriate, see recommendation 1.2.28), offer a planned caesarean birth for women requesting a caesarean birth (2011, amended 2021).

This shift in approach to caesarean delivery in the United Kingdom can also be seen in the move from caesarean rates as a metric for the performance of hospitals.^14^ The express policy in the United Kingdom is notable considering the comparatively slower development of legal precedent regarding the standard of care pertaining to consent.^1^ Despite the established duty in Rogers v Whittaker, some 20 years prior, the United Kingdom had not advanced the standard of care required of medical practitioners with respect to the provision of information until the decision of Montgomery v Lanarkshire Health Board UKSC 11.^15^ Montgomery concerned the failure of an obstetrician, Dr McLellan to adequately inform her patient Mrs Montgomery of the risk of shoulder dystocia during delivery, despite the 9%–10% risk of it occurring. Dr McLellan gave evidence that despite this high risk, she did not discuss shoulder dystocia as she considered the risk of harm to be small and that in her experience, most women will choose a caesarean in the circumstances. Dr McLellan further stated:

[I]f you were to mention to any mother who faces labour that there is a very small risk of the baby dying in labour, then everyone would ask for a caesarean section, and it’s not in the maternal interests for women to have caesarean sections.^15^

The failure of ethics in this statement is striking. First, as we will argue, the risks and benefits associated with caesarean section are different to those of vaginal birth. These require an evaluation by the delivering woman herself, to assess which risks she prefers to take, and which she prefers to avoid. But second, even if it was clear that vaginal delivery was in the maternal interests, it is also important to take into account the pregnant woman’s role as advocate of her future child’s interests.

How these should be weighed against maternal interests is again a decision the woman should be supported to make.

The duty owed to patients to be informed of risks, is reflected in Montgomery, at 84, the Court found:

Furthermore, because the extent to which a doctor may be inclined to discuss risks with a patient is not determined by medical learning or experience, the application of the Bolam test to this question is liable to result in the sanctioning of differences in practice which are attributable not to divergent schools of thought in medical science, but merely to divergent attitudes among doctors as to the degree of respect owed to their patients.

Despite the express inclusion of MRCS in the NICE Guidelines, there has been debate as to whether this has equated to providing women the choice in practice or whether pregnant women are still subjected to the elective caesarean lottery.^16^

It is acknowledged that for each pregnant woman, individual circumstances, such as age, weight and medical history can influence their risk profile and be relevant in assessing the risks and benefits of birthing options.^17^ This analysis, focused on the homogenised risks of vaginal versus caesarean birth, demonstrates that for a pregnant woman, on balancing the risks and benefits of vaginal birth, MRCS may be a reasoned choice as opposed to one made in fear.^18^

Strictly speaking, one does not need to obtain consent from a woman for her to have a vaginal delivery. Vaginal delivery itself does not necessarily require any medical intervention. It will occur, in some form, even if the woman and others do nothing. As such, there can be no legal requirement to obtain consent for vaginal delivery to go ahead, and no way in which a woman can refuse consent for vaginal delivery and, in doing so, prevent it from happening. This does not, however, mean that it is acceptable for medical professionals to allow women to proceed through vaginal delivery unaware of the risks it involves and alternatives available. Medical professionals have a duty of disclosure, even when not directed at consent for an intervention, in order to facilitate autonomous choices.

A potential problem in medicine is where ‘natural’ processes, like vaginal delivery, are seen as not requiring discussion because they represent a default and would occur if nothing was done. But once there is the power to intervene, responsibility requires that the reasonably foreseeable effects of all alternatives, including doing nothing, are discussed.

## Risks of childbirth

### Vaginal

In Australia, the maternal mortality ratio was 8.5 deaths per 100 000 women in 2016.^19^ Leading causes of maternal death are pre-eclampsia, obstetric haemorrhage, sepsis and cardiovascular problems.^20^ A vaginal birth or a trial of labour can range from a birth requiring no external or medicative assistance to the use of interventions such as forceps or vacuum extraction and the use of analgesic medications. Further despite all efforts, a caesarean section can still be required following a trial of labour.

The risk of perineal tearing, and its associated complications, is an obvious concern for pregnant women. Approximately, 3% of all women who give birth vaginally in Australia suffer a third or fourth-degree perineum tear,^21 22^ for first-time mothers, this rate is increased to represent 5%^22^ of all vaginal births. A third-degree perineal tear involves an injury to the anal sphincter, whereas a fourth-degree tear also involves damage to the anal mucosa (or lining). Without repair, the tears can result in long-term consequences such as ‘continued perineal pain, faecal incontinence, painful sexual intercourse, reduced quality of life and depression’.^22^ The risk of perineal tears increases to 7.3% with instrument-assisted vaginal births. There has been a rise in reported rates of severe perineal trauma, but this may reflect an increase in diagnosis as opposed to changes in clinical care.^23^

One risk of pregnancy and perineal tearing is the potential to increase the chance of urinary incontinence shortly following birth and long term. A study found that at 12 years following childbirth, 52.7% of women reported urinary incontinence at some point, 23% reported weekly occasions of incontinence and 5.4% reported daily occurrences.^24^ Similarly, childbirth is associated with faecal incontinence. Women suffering from faecal incontinence report having a reduced quality of life, utilised pads, plugs or constipating medicine as a result. Incontinence is greatly reduced in women who have exclusively had caesarean births, indicating the procedure has a protective element.^24^

Vaginal birth increases a woman’s risk of pelvic organ prolapse to 29% as compared with 5% for women who have only had caesarean births.^25^ This risk increases further with the use of forceps in vaginal delivery.^25^ An obstetric fistula is a less common risk of vaginal birth in Australia, associated with obstructed labour occurring in two in 100 000 women.^25^

### Caesarean section

Caesarean births are not without complication, involving a ‘major, open-abdominal procedure, often performed in an emergency setting’.^26^ As with any surgery, risks include blood loss, wound infection, deep vein thrombosis, pulmonary embolus, damage to adjacent organs necessitating corrective surgery, slower recovery and risks associated with anaesthesia such as vomiting, nausea, postdural puncture headache and low blood pressure.^27^

RANZOG guidelines provide that elective caesareans have a 7% risk of complications, compared with 16.3% for emergency caesareans and 12.9% for instrumental vaginal deliveries.^5^ Postoperative sepsis or puerperal fever is an important risk, at a rate of 9.7% for emergency procedures and 6.8% for planned caesareans.^26^ Caesarean delivery is associated with a postpartum haemorrhage rate of 5%, but this rate drops to 1.1% for planned caesarean sections.^26^ Elective caesareans are also associated with a reduction in breastfeeding rates initially postbirth, but not once feeding has commenced.^28^

Relevantly for future or subsequent births, caesareans are associated with an increased placenta accreta—where the placenta does not come away from the muscle of the womb after birth—which compounds each subsequent pregnancy. It may be associated with significant maternal mortality and morbidity, including massive haemorrhage requiring emergency hysterectomy.^5^ Women who have a caesarean delivery for their first pregnancy are also less likely to have any subsequent pregnancies.^29^ Caesarean delivery is commonly associated with the development of abdominal adhesions, which are correlated with their own morbidities, such as chronic pelvic pain, secondary infertility and bowel obstruction. The incidence of abdominal adhesions increases with each caesarean procedure and also increases the risk that subsequent surgical procedures will be required.^30^

### Neonatal risks

According to the RANZOG Guidelines, there is a lack of reliable and accurate data on the effect of all birthing options, limiting understanding of the respective risks.^5^ A Western Australian study discovered rates of hypoxic ischaemic encephalopathy (a complication where inadequate blood flow reaches the baby’s brain) were significantly lower with elective caesarean sections than alternative modes of birth.^5 31^ There is the potential for a planned early caesarean delivery to be preventive for injuries to the baby, as cerebral palsy and Erb’s palsy are ‘unequivocally greater after vaginal birth’ with rates reported at up to 0.3%.^5^

The Guidelines provide that ‘caesarean delivery without labour is associated with an increased risk of neonatal respiratory complications’ (rapid breathing).^5^

While RANZOG guidelines state that the risk of fetal injury is low with caesarean delivery, there is still the possibility of injuries such as skull fractures, intracranial haemorrhage, brachial plexus palsy or cervical spine, spinal cord and/or vertebral artery injury.^32^ Complications and injury can still arise in cases of a very large fetus, but the incidence is much less than with vaginal birth.^32^ There is also evidence that caesarean births without labour (not necessarily as a result of maternal request) increase short-term neonatal respiratory problems (including transient tachypnea) and childhood infections until 5 years of age.^5 6 33^

## The ethical case for consent

As discussed, vaginal birth has an increased risk of perineum tears, incontinence and the use of vacuum or forceps. It also poses higher risks of hypoxic brain injuries and nerve damage to the baby. Moreover, attempting vaginal delivery still has the potential for a delivery by emergency caesarean. This occurs around 18% of the time for nulliparous (first time) mothers.^34^ Emergency caesareans are associated with a higher risk of infections and complications.^5 26 35 36^ Meanwhile elective caesareans have risks associated with surgery such as deep vein thrombosis, pulmonary embolus, organ damage and increased risk of placenta accreta in subsequent pregnancies. In addition to the physical risks outlined above, there is increasing recognition of the risk and impact of birth trauma. The Australian Birth Trauma Association states that birth trauma can be psychological or physical. They provide the following description:

The psychological trauma can be the result of an extreme disconnect between a woman’s expectations of what would happen and the shock of what actually occurred. It may also relate to feelings of loss of control and a sense of not having a ‘voice’ in the face of authority, and unexplained interventions, as well as the physical damage.^37^

Considering the multitude of respective risks of vaginal birth, emergency caesareans and elective caesarean deliveries and the long-term implications for mother and baby, it is reasonable to ask why women are not routinely provided with the risks and benefits of all methods of birth prior to delivery.^2^ ^7 38^

Informed consent requirements are designed to ensure that patients are made fully aware of the options available to them regarding their medical care. They can serve to protect patients from the paternalistic actions of healthcare professionals, such as the behaviour of Dr McLellan in not discussing the risk of shoulder dystocia with Mrs Montgomery for fear that she would make the ‘wrong’ choice in electing for a caesarean section.^39^

They may alternatively—or additionally—act as a waiver, signalling that the patient permits her medical team to violate her bodily integrity in a way not ordinarily allowed.^40^

Where a decision has been made to proceed with the pregnancy or a miscarriage has not occurred, childbirth is a predictable consequence of pregnancy. Many deliveries will involve interventions from medical teams, whether that be vaginal examinations to assess the progression of labour, midwives providing pain relief during a home birth, the use of forceps in a hospital setting or surgery in the form of caesarean section. The location of delivery or birth setting, for example, home birth, midwifery or obstetric unit combined with a woman’s pregnancy risk profile can influence the likelihood of adverse events or use of interventions.^41^ Further medical care may follow delivery—stitches may be required to repair tears and episiotomies, drugs administered to reduce haemorrhage risk or treatments for the newborn baby. Many of these interventions are intrusive and some carry with them significant risks, including acute and chronic pain and disabling and disfiguring effects.

Although it is unpredictable how labour will proceed, it is possible to prepare women for a number of eventualities, particularly those which are reasonably likely to occur. For instance, the vast majority of women in New South Wales give birth in a hospital, including 23% of nulliparous mothers who plan a home birth.^42^ Assisted vaginal delivery (where forceps or vacuum extractor are used) is also more common for first-time mothers in Australia, where rates indicate that forceps are used for 1 in 10 births and vacuum extraction for approximately 1 in 7 births.^43^ In Australia, nearly 1 in 5 women giving birth for the first time will have an emergency caesarean section.^34^ Thus, giving birth in a hospital (with the attendant intimate examinations), having an assisted delivery and having an emergency caesarean section are all eventualities that are reasonably likely to occur, along with their associated morbidities. Given that labour is likely to be a time of high stress, where the mother may be in significant pain, discussing risks and benefits of different birthing interventions for the first time at this point is undesirable.

This suggests that the process of informed consent could be beneficially applied prior to labour. As always with informed consent, the patient is free to withdraw consent at any point and to change their decision regarding how treatment will proceed. In the remainder of this section, we reflect on how ethical values interact with decisions surrounding mode of delivery.

### Autonomy

A primary concern when considering medical decision-making and appropriate information sharing is the need to ensure that patient autonomy is respected. Proponents of ‘natural birth’ provide examples of paternalistic medical interventions and experiences of women who have had traumatic births in which they have not had their autonomy or rights respected. They warn of the exploitation of statistical and risk information as a tool to influence women’s decisions about their care^44^ and fear a slippery slope, whereby pregnancy is further pathologised, creating additional fear, pressure, conflict and loss of control among women.^45^ This leads to a blanket suspicion of any and all medical interventions in childbirth, and a preference for ‘natural’ deliveries (ie, vaginal, in a non-medical setting, often with no pharmaceutical pain relief or assistive technologies). Outside the natural birth movement, authorities such as WHO also seek to reduce the rates of caesarean sections, suggesting they should be performed only 10%–15% of the time.^46 47^ There is concern that, where caesarean section rates exceed this it is driven by practitioner rather than patient preferences, perhaps due to the convenience of being able to schedule births or a desire to mitigate risks.^48^ Thus, autonomy may be used as an argument against policies that might increase caesarean section rates.

Ethically, there is a difference between having a right to refuse a certain intervention, and having a right to demand some treatment.^49 50^ Previously, a lack of evidence showing a net benefit of caesarean sections spoke against a right to demand such a procedure, yet a more holistic consideration of how women might reasonably weigh the outcomes of different modes of birth for both themselves and their future child means the question of net benefit is not so clear.^13^

We suggest it is implausible that a concern for autonomy should direct us to withhold risk information regarding trial of labour (both relating to the woman and the fetus) from pregnant women.^18^ Given the risks described earlier, a rational woman might well determine that she is willing to accept the harms associated with surgery (based on personal experience or impersonal data) in order to avoid the harms associated with vaginal delivery, such as third-degree or fourth-degree tears, urinary or faecal incontinence and the risk of extreme acute pain. Alternatively, she may be prepared to sacrifice her own interests to avoid even a small risk to her baby. Such altruistic preferences should be respected. There is an excellent case for considering childbirth a preference-sensitive decision: the risks and benefits of different modes of delivery are not decisive in terms of which will best promote the interests of the mother and baby, and it will depend on each individual’s values and preferences.^51^ It is perhaps instructive that obstetricians and other healthcare professionals seem to prefer caesarean births relative to the general population.^52^,^54^

Furthermore, medicalised birth is not inherently incompatible with women’s autonomy.^18^ Women may choose a medicalised birth and prefer their healthcare team to take control over their delivery, meaning the medical management of birth can be experienced as empowering for some women.^45^ The use of terms such as ‘normal’, ‘natural’, ‘right’ or ‘wrong’ to describe modes of delivery or procedures is also not supportive of beneficial power and a pregnant woman’s ability to maintain control over her perception of the world.^55^

In accordance with standards of shared decision-making, the respective risks associated with different modes of birth should be explicitly discussed with the pregnant woman such that she can plan the birth she prefers. Moreover, the current legal landscape, as defined by *Rogers v Whittaker*, requires that informed consent is given for any medical interventions that occur during assisted delivery, including communicating information about risks likely to be material to the mother. As already argued, prior discussion of the harms and benefits of different modes of birth would ensure women are enabled to make informed choices regarding their birth options and can consent to any interventions likely to be encountered (recognising that consent will need to be resought and may be removed at the time any interventions occur).

### Neonatal well-being

The mother is not the only agent affected by the risks inherent in childbirth. It is well recognised *‘in both medical ethics and law that patients with capacity can refuse treatment, even if doing so is against their interests’*.^56^ Despite this well-established principle, the moral permissibility of forced medical care on a pregnant woman when the unborn fetus is at risk is subjected to continued debate.^56 57^ As discussed, different modes of delivery are associated with different risks to the child as well, and it is appropriate to consider their interests when weighing different birth options. Occasionally, a mother’s preference regarding childbirth may conflict with the interests of her child, as can be seen in ethical debates concerning home birthing or the moral permissibility of forced caesareans.^56 58^ In such circumstances, where the birthing choice carries an additional risk of life-long disability for the child, an ethical challenge arises in the form of balancing the pregnant woman’s autonomy and right to self-determination, and the fetus’s interests in not being harmed. On one hand, ensuring a woman’s autonomy, which on pregnancy, she does not become a means to an end or a restrained ‘ideal baby-making machine’.^59^ However, women will typically be highly motivated to ensure that minimal harm comes to their child during delivery.^60^ Assuming that the pregnant woman intends on being responsible for the child’s care following birth and, therefore, has a *secondary benefit* in the health of the fetus, it also cannot be assumed that the two interests are mutually exclusive.^61^ Thus, ensuring that women are informed of the ‘material risks’ associated with vaginal and caesarean births should support them to make decisions in the interests of their child as well as themselves.

## Objections

### Requiring consent is conceptually confused

A principal objection might be that planned vaginal delivery just is not the sort of thing to which one can consent. Or rather, it is not the sort of thing to which one can *refuse* consent: it is a natural consequence of pregnancy, not an optional medical intervention.^62^ Furthermore, there are concerns that women are pressured into medical interventions during delivery and that childbirth has become routinely medicalised.^63^ Encouraging women to view vaginal birth not as the default mode of delivery but as one alternative, complete with attendant risks, might be seen as a further shift towards medicalised childbirth.

While it is true that there is something odd about adopting processes of consent for a planned vaginal birth, we feel that the potential benefits could outweigh the conceptual drawbacks. First, as discussed, it would provide an important (and much needed) opportunity for pregnant women to discuss the potential risks (and benefits) of different modes of childbirth. This would be at a point in time prior to labour when they are able to consider their preferences regarding different risks and discuss these with their healthcare team, as opposed to rushed decisions at *particular times*.^18^ This may enable some women to make decisions ahead of labour, which mean they experience a childbirth that fits better with their preferences (eg, seeking a caesarean section) and avoids outcomes they particularly wish to avoid.

Second, the mere ‘inevitability’ of childbirth does not render consent procedures irrelevant. As described by Manson and Neil, providing informed consent constitutes a waiver, permitting healthcare professionals to take actions that would ordinarily be a violation of one’s bodily integrity.^39^ Act of giving informed consent changes the meaning of those actions from violations and injury to remedy and care. As Kingma argues, there are characteristics of maternity care that ‘*increase* the need for explicit attention to, and respect for both autonomy and rigorous informed consent processes’.^61^ Giving women the opportunity to affirm their willingness to undergo interventions associated with vaginal delivery is, thus, a valuable process of permission-giving.

One particular concern is that the language of ‘normal’ and ‘natural’ birth encourages pregnant women to assume that opting for a vaginal delivery is *very likely* to result in a birth with no medical interventions. As discussed, interventions such as vaginal exams, instrument deliveries, episiotomies and emergency caesareans are relatively common. In choosing a trial of labour, therefore, the pregnant woman is effectively (though perhaps not knowingly) choosing to place herself in a position where she is likely to be asked to consent to any number of these interventions. It may be very difficult (not to mention unwise) to refuse instrumental delivery or emergency caesarean once one is partway through a difficult labour. Hence, the importance of being given the opportunity to consider, when forming a birth plan, which risks one is willing to be exposed to, and which interventions one is willing to undergo.

### Risk information is distressing

A second objection might be that highlighting the risks associated with (vaginal) childbirth could cause additional distress. A German study from 2003 suggested that women labelled as ‘at risk’ in pregnancy may suffer psychologically.^64^ Medical professionals’ understanding of risk is claimed to be based on specialised training and knowledge, in conjunction with personal experiences and value. In contrast, pregnant women’s is considered ‘contextual, individualised and embedded in their social environment and individual lives’.^64^ Adopting a medical risk-based model of childbirth may emphasise clinical risks and neglect the positive aspects of childbirth—for instance, as a spiritual and empowering experience for mothers.

It may, indeed, be distressing to be informed of the risks associated with a trial of labour, and to face the prospect of perineal tearing, incontinence and severe pain. Yet to allow the unpleasantness of being informed—truthfully—of one’s medical risks to justify withholding relevant information from people is dangerous. This was the justification provided by Dr McLellan in the Montgomery case: Dr McLellan was concerned that information regarding the size of the baby was causing Mrs Montgomery distress, and, hence, no scan was conducted at 38 weeks; the (high) risk of shoulder dystocia was considered likely to provoke a request for a caesarean section and, hence it was not discussed.

Compassion is a laudable quality in a medical professional, and a desire to avoid unnecessary suffering is understandable. This should not, however, extend to misleading a patient (including via omission) regarding the material risks she faces.

In the Montgomery case, it appears that Dr McLellan was aware of the materiality of the risks of shoulder dystocia to her patient, since she predicted that, if such risks were made known to Mrs Montgomery, she would have requested a caesarean section, and Dr McLellan judged a caesarean section to ‘not be in the maternal interests’. Yet a patient’s right to make decisions not regarded in their interests by a medical professional is protected by medical ethical guidelines and law. So long as patients meet the requirements of competence to decide on their treatment, they should be permitted to choose which treatments they consent to and which they refuse. This is only possible if patients are provided with the relevant information in order to make those decisions.

Earlier reviews and studies of the psychological consequences of caesarean deliveries noted ‘increased fear during labour, loss of positive self-esteem and body image, feelings of failure and self-blame and post-traumatic stress’.^65^ The description of vaginal birth as a ‘normal’ or ‘natural’ birth may contribute to women’s feelings of failure or disappointment in their birth experience, as by inference, their birth was thereby ‘abnormal’ or ‘unnatural’. Furthermore, a woman’s perception of her control or absence thereof, during the birth can also contribute to potential psychological consequences of a caesarean delivery.^65^ One suggested method of reducing this risk is to ‘provide women with information about caesarean delivery prenatally and help them develop realistic expectations about childbirth’.^65^ Providing more information to women may assist in the fear and anxiety they feel at the prospect of losing control and autonomy as they approach labour and birth.^66^ As such, it is at least plausible that additional information provision will not ultimately result in more, but rather less, distress.

### Cost

Finally, cost considerations might speak against seeking consent from women for vaginal birth. The time needed to inform women of the risks of vaginal delivery would create additional costs to the healthcare system. Furthermore, the cost of a vaginal birth is estimated at $9089 in Australia compared with $14 086 for a caesarean delivery.^67^ However, cost estimates from Canada suggest caesarean delivery (prior to onset of labour) is comparable to vaginal delivery, and cost-saving relative to assisted vaginal delivery.^68^

Nonetheless, if seeking consent for planned vaginal delivery and routinely providing information regarding the risks of all modes of birth were to result in more caesarean sections, then this could plausibly make maternity care more expensive. It is not clear that pregnant women should be offered a medically unnecessary procedure when an acceptably safe, more resource efficient alternative is available. This is compounded by the likelihood that a caesarean section makes it more likely that any subsequent deliveries will be by caesarean section as well. Highlighting the risks of trial of labour by conducting informed consent procedures could increase MRCSs and lead to a slippery slope, whereby the majority of births are by caesarean section and vaginal delivery is a rarity.^69^

Any additional costs should be weighed against benefits that might be gained later in the birthing process or after birth. For practitioners and healthcare providers, increased elective caesareans may decrease insurance premiums^53^ and may assist time management and resourcing allocations, although this would need to be measured against the increased length of stay for mothers’ postsurgery. On the other hand, the decreased rates of permanent neonatal injuries and disabilities that could occur with higher rates of caesarean delivery may counterbalance the time cost incurred in the consent process. Once the cost of a potential stay for babies in the NICU (more frequent with vaginal birth),^68^ along with the long-term cost of prolapse, is calculated, the argument that caesarean deliveries cost more is not true.^70 71^

It is, therefore, unclear that routinely engaging in shared decision-making processes and seeking consent for different modes of childbirth would be additionally costly. Even if the absolute cost was to increase, it is unclear that it would, all things considered, be cost inefficient. Given the benefits of providing additional risk information (in terms of respecting women’s autonomy and facilitating better decision-making regarding their mode of delivery), any additional expense may be worthwhile in terms of the pay off.

Indeed, even if caesarean sections were more costly, and should not be funded within a public healthcare system, women should still be informed of the risks of planning a vaginal delivery and enabled to make their own decision whether to pursue alternatives privately.

## Concluding remarks

We have argued that there is currently a divide between ethical care and current practice in Australian obstetric care. We suggest this could, in part, be remedied by routinely providing information to women on the risks of all methods of birth at a predetermined stage of pregnancy, early enough to allow sufficient consideration of the options available to birthing women and to consider their willingness to consent to interventions (and the corresponding risks) associated with different modes of birth. Vaginal delivery is a complex course, with many forking paths (some associated with interventions requiring consent), and multiple possible outcomes, including severe complications. There is an alternative at the outset, elective caesarean and multiple alternatives as childbirth progresses. Women need to be informed of the harms and benefits of alternative courses of action, so they may act according to their relevant values, particularly their own health and that of their future child.

If childbirth is analogous to running a marathon, only discussing potential risks *during* the race is not conducive to motivation and completion of the event. Furthermore, a complete absence of information at the outset fails to adequately prepare the marathon runner for what lies ahead. The current timing of the disclosure of the risks of interventions such as forceps or caesarean delivery, after a delayed labour or due to an emergency, does not provide an opportunity for women to make a clear and reasoned decision. Early discussion will not only facilitate better appreciation of risks and ability to judge preferences but also allow women the opportunity to consider their options and prepare for coming eventualities. The provision of accurate information will also help women to form reasonable expectations regarding delivery itself and its after effects. This could increase women’s empowerment and decrease the incidence of birth trauma and the numbers of women silently suffering from post-birth complications.

There is currently an unjustified value judgement inherent in the promotion of ‘natural’ birth. This contributes to women’s sense of failure in an unpredictable and at times traumatic event. Childbirth is described as ‘one of the most pivotal moment’s in a woman’s life’,^72^ which it ‘allows women to fully appreciate the power of the birthing body’ or that it is a demonstration of their commitment to motherhood.^44^ The romanticisation of birth unrealistically assumes that for all women, birth is an authentic empowering experience.^44^ It assumes that for all women, the physical birth of their child is the achievement as opposed to the result: having a child. It assumes that all women will have an equal ability to cope with an adverse outcome, that irrespective of their personal history, a ‘natural’ birth is in their interest. We can begin to address the damage done by these unrealistic expectations of childbirth by ensuring that unassisted vaginal delivery is not valorised as the optimum (nor expected) outcome for all women, and a trial of labour is instead presented accurately, with its attendant risks and their likelihood of occurring.

Women may choose a birthing option, which they judge to be in their interests despite their doctors judging it to be against their interests. They may choose options which are against their interests for the sake of their future child, but these are their choices to make. The only reason not to offer MRCS as an alternative to normal vaginal delivery is on the basis of distributive justice. There is no compelling reason to withhold relevant information about the benefits and risks of planning a vaginal delivery and the alternatives.

## Data Availability

No data are available.

## Footnotes

It is worth noting that, although the NICE guidelines recommend provision of caesarean section upon maternal request, this does not require—nor has it resulted in—blanket provision of maternal request caesareans across England.

It is hard to establish that healthcare professionals rarely or never volunteer information regarding elective caesareans to pregnant women. It is, however, our understanding that this is unusual. First, women are not typically able to access elective caesareans via public healthcare in Australia, so discussing them with women would be somewhat surprising. Indeed, many public hospitals have regulations against caesarean delivery on maternal request.^7^ Second, a number of studies indirectly establish the lack of information regarding elective caesarean by describing, for instance, women’s dissatisfaction with the information they were given regarding their birth options.^38^
